# Supplementary material for: Cardiometabolic multimorbidity and associated patterns of healthcare utilization and quality of life: Results from the Study on Global AGEing and Adult Health (SAGE) Wave 2 in Ghana
Source: PLOS Glob Public Health. 2023 Aug 16;3(8):e0002215. doi: 10.1371/journal.pgph.0002215 (PMC10431646; doi:10.1371/journal.pgph.0002215)
Supplement: S2 Table — (PDF) [file pgph.0002215.s002.pdf]

S2 Table: Comparison between latent class models

Number of latent classes	CAIC	BIC
1	22909.5	22976.0
2	22498.0	22364.9
**3**	22043.1	22254.8
**4**	**21961.0**	**22233.2**
5	21941.3	22280.0

Note: Boldface type indicates the selected model. BIC Bayesian Information Criterion, CAIC consistent Akaike Information Criterion.
