# Supplementary material for: Cardiometabolic multimorbidity and associated patterns of healthcare utilization and quality of life: Results from the Study on Global AGEing and Adult Health (SAGE) Wave 2 in Ghana
Source: PLOS Glob Public Health. 2023 Aug 16;3(8):e0002215. doi: 10.1371/journal.pgph.0002215 (PMC10431646; doi:10.1371/journal.pgph.0002215)
Supplement: Attachment — Submitted filename: Responses to reviewers comments.docx [file pgph.0002215.s003.docx]

**Response to the editors and reviewers:**

We thank the editors and reviewers for their comments to improve the manuscript for publication.

**Journal Requirement Queries**

1. Please ensure that Funding Information and Financial Disclosure Statement are matched.

Funding Information and Financial Disclosure Statement have been matched

2. In the Funding Information you indicated that no funding was received. Please revise the Funding Information field to reflect funding received.

Thanks for this observation. The information has been added.

3. Your manuscript is missing the following sections: Introduction. Please ensure these are present, and in the correct order, and that any references to subheadings in your main text are correct. An outline of the required sections can be consulted in our submission guidelines here:

<https://journals.plos.org/globalpublichealth/s/submission-guidelines#loc-parts-of-a-submission>

The introduction section has been added. Previously this was titled “Background”

**Editor’s comments**

4. On what basis were the non-cardiometabolic chronic conditions selected for inclusion in this study?
Why were cataracts included?

Many thanks for this observation. Unrelated or “discordant” conditions (e.g. cataract arthritis, chronic respiratory diseases and depression) often coexist with cardiometabolic diseases although they may not share the same management plans or pathophysiology [1]. In contrast, cardiometabolic multimorbidity may also be “concordant” if the co-occurring conditions represent parts of the same overall pathophysiologic risk profile and have the same disease management plan ie hypertension, diabetes, CVD, hypercholesteremia. In the current study, we analysed the clustering of cardiometabolic diseases including angina, stroke, diabetes, and hypertension with unrelated conditions such as asthma, chronic lung disease, arthritis, cataract and depression

5. Lines 124-126: Please check if this sentence is correct. Why were participants deemed to have a condition if they were ‘screened negative’?

Thank you very much for this observation. This has been revised. In lines 129-136 (page 6), we have indicated that self-reported history of diagnosis by a healthcare professional was extracted for cardiometabolic diseases comprising angina, stroke, diabetes, hypertension and other conditions such as arthritis, asthma, chronic lung disease, depression, and cataract. The WHO symptomatology algorithms [2-4] were used to screen for angina pectoris, arthritis, asthma, chronic lung disease, and depression. Online supplementary file 1 shows the details of the symptomatology algorithms. Physical measurements comprised screening for blood pressure (BP). Hypertension was defined as systolic BP ≥ 140 mmHg and/or diastolic BP ≥ 90 mmHg or previous diagnosis of hypertension by a professional health care provider and/or being on hypertensive therapy [5].

6. Please present Table 1 together with the overall data by men and women as well together with p-values for gender differences.

Table 1 showing the overall data by men and women as well together with p-values for gender differences has been added (lines 201, page 9)

7. How do the faith-based/charity organisations differ from public healthcare facilities in terms of fees charged, etc.?

Thank you very much for this observation. Indeed, the cost of care is very important in understanding the burden of multimorbidity among patients who seek care from public, private and faith-based facilities. Under the limitation sections for this study (lines 321-326 (page 16)), we have indicated that this study assessed the association of different cardiometabolic multimorbidity combinations with the frequency of outpatient visits and hospitalization in Ghana. However, the nature of outpatient visits or hospitalization such as routine care or emergencies was not explored. Furthermore, the association of multimorbidity clusters with the cost of care were not investigated. Thus future studies on the economic burden of different cardiometabolic multimorbidity combinations on are needed.

8. Table 2: Please define the multimorbidity clusters in the table or footnotes - what does minimal multimorbidity refer to? Tables need to be standalone without referring to the details in the main text.

Thanks for this observation. The multimorbidity clusters have been defined as footnotes in table 2.

**Reviewers' comments:**

**Reviewer 1**

1. The article classified multimorbidity in older adults and assessed the association between these classes with healthcare utilization and quality of life. The results can affect planning of healthcare policies and interventions which will improve healthcare outcomes among older adults.
Even though the manuscript appears sound, the authors should clarify if the following terms refer to the same class to avoid confusion as they were used interchangeable. They should also clearly define these terms in the explanatory variables.
Minimal multimorbidity ( line 34, table 2, line 199, 214, 223,232, 324)
Minimal cardiometabolic multimorbidity (line 179, 211, 216)

Thank you very much for this observation. This has been revised. The multimorbidity clusters were named based on their unique dominant chronic diseases (line 126, page 6). Our findings show four distinct patterns of multimorbidity: relatively “healthy class” with minimal chronic diseases: abdominal obesity: cardiometabolic and arthritis class comprising participants with hypertension, diabetes, stroke, abdominal and general obesity, arthritis and cataract; and cardiopulmonary and depression class including participants with angina, chronic lung disease, asthma, and depression (lines 201-207 (page 9-10)).

2. The authors stated that one of their strength was screening for chronic diseases which was based on objective measures comprising direct physical measurement of BP, symptomatology algorithms and self -reports (line 249) However they also stated that one of the limitations of the study was screening for chronic diseases which were partially based on self- report.(lines 254). This looks contradictory

Thank you very much for pointing out this. This has been revised. Indeed one major strength of this study was the fact that screening for obesity, hypertension, angina pectoris, arthritis, asthma, chronic lung disease, and depression was based on objective measures comprising direct physical measurement of anthropometrics, BP, symptomatology algorithms and self-reports (lines 311-314 (page 15). However, under the limitation section (lines 318-320 ((page 15), we have indicated that the screening questions for diabetes, stroke and cataract were based on self-reported history of diagnosis. This may have resulted in the underestimation of the true prevalence of chronic diseases.

3. In the conclusion, the sentence on line 276 – 277 should be rephrased to make the message clear.

Thank you very much for this observation. This has been revised. The findings of this study show that cardiometabolic multimorbidity among older persons in Ghana cluster together in distinct patterns that differ in healthcare utilization and QoL. This evidence may be used in healthcare planning and development of appropriate clinical guidelines for the management of cardiometabolic multimorbidity. Our findings form the basis for, future research on the aetiology and pathogenesis of discordant multimorbidity clusters, and improved policies to address healthcare access and QoL for older persons living with cardiometabolic multimorbidity in sub-Saharan Africa. (Lines 341-346 (page 16-17)).

**Reviewer 2**

Thank you for the opportunity to review this compelling manuscript. This work represents a meaningful contribution in an understudied area using innovative statistical techniques to identify multimorbidity clusters of potential relevance to health services delivery in a global population.

There are a few areas of major improvements to this manuscript.

1. Firstly, the data used in this study are from 2015. While SAGE Wave 3 data from 2018-2019 are still pending, it may potentially be valuable to wait to provide an update on this cluster analysis with newer data, given that the data represented are 8 years old with the prospect of a newer data set. The newer data may provide a more meaningful representation of the current status of the population’s multimorbidity and needs.,

Thank you very much for this important suggestion. This has been added under the limitation section (line 329-333(page 16)). The WHO SAGE data used in this analysis were collected in 2015, and rapid changes in health and socioeconomic circumstances in Ghana are likely to have affected the burden of chronic diseases and quality of life in the last 8 years. Nevertheless, our findings are based on the most recent data we could access and act as a baseline with which to compare future studies on the burden of multimorbidity on healthcare utilization and quality of life in Ghana.

2. Additionally, it is to be expected that patients with a higher disease burden would receive more outpatient and inpatient services. It would strengthen this analysis to include data on what kind of outpatient visits and inpatient hospitalizations patients who group into clusters received. For example, were there a greater degree of urgent outpatient visits recorded for non-routine reasons such as medication refills, but rather for exacerbations of pathologies? This would strengthen an understanding of what kinds of services are most needed by this population.

Thank you very much for pointing out this. Understanding the patterns of multimorbidity is important for planning health system capacity and response. The current study assessed the association of different cardiometabolic multimorbidity combinations with the frequency of outpatient visits and hospitalization in Ghana. However, under the limitation section (lines 318-321, page 15-16), we have indicated that the nature of outpatient visits or hospitalization such as routine care or emergencies was not explored. Furthermore, the association of multimorbidity clusters with the cost of care were not investigated. Thus future studies on the economic burden of different cardiometabolic multimorbidity combinations are needed.

3. The authors also posit that the clustering of cardiopulmonary diseases may be explained by sociodemographic factors such as smoking, however, data on participants’ smoking history, access to food support, etc. are not provided. Inclusion of this data would strengthen the causal connection, although it may be beyond the scope of what was collected in this study. Regardless, mentioning these variables in the analysis and discussion of why or why not it was included would be valuable.

Thank you very much for this important observation. We have added a detailed description of the sociodemographic distribution of the multimorbidity classes identified in this study (Table 3, page 10). We have previously published a manuscript on the behavioural risk factors associated with cardiometabolic multimorbity in Sub Saharan Africa [6]. The focus of the current study therefore, was to assess the association of different cardiometabolic multimorbidity combinations with healthcare utilization and quality of life. Nevertheless, possible explanations for the multimorbidity clusters from existing literature have been highlighted under the discussion section (Lines 277-286 (page 10)).

4. Additionally, hypertension is included in class 2 (hypertension and arthritis) and 3 (hypertension, angina, chronic lung disease, and asthma) of the cluster analyses. Given the dual appearance of hypertension within these clusters, it would be important to show any potential interaction between these clusters and whether there is any overlap or competing effects from cluster type and visits/hospitalizations.

Thank you very much for this observation. Our findings show four distinct patterns of multimorbidity: relatively “healthy class” with minimal chronic diseases: abdominal obesity: cardiometabolic and arthritis class comprising participants with hypertension, diabetes, stroke, abdominal and general obesity, arthritis and cataract; and cardiopulmonary and depression class including participants with angina, chronic lung disease, asthma, and depression. (Lines 201-210 (page 9-10)). Further analysis, revealed no significant interaction between the latent classes of multimorbidity.

*Minor revisions include:*

*- Proofreading needs*

5. Authors mention the phenomenon of discordant multimorbidity but do not discuss the role of depression in relation to the multimorbid conditions mentioned. Additionally, the authors mention integrated care interventions that would benefit patients with multi morbid conditions, but the paper would be strengthened by inclusion of examples of these interventions.

Thank you very much for pointing out this. In lines 278-290 (page 14), we have indicated that the mechanisms that underlie the clustering of cardiopulmonary diseases and depression are not definitive. However, there is strong evidence linking inflammatory markers to both depression and cardiovascular diseases [7], but why these links exist remains unclear. Systematic reviews conducted by Mullerova et al. [8] and Prados-Torres et al. [9], identified inflammation, stress processes, hypoxia, and environmental risk factors such as air pollution and smoking as the leading risk factors for the clustering of cardiopulmonary diseases such as hypertension, angina, chronic lung disease and asthma [8, 9]. Similarly, our previous study on the patterns of cardiometabolic multimorbidity in sub-Saharan Africa identified the clustering of physical inactivity and obesity as one of the leading risk factors for cardiometabolic multimorbidity

**Reviewer 3**
There are several methodological issues.
1. The authors state “Detailed descriptions of sampling methods and data collection procedures have been previously published (26)”, “Data used in the current study were collected using interviewer-administered structured questionnaires (26)”. “Detailed information on the study tools has been published (28).”The data set and study design used for the study are not available at the cited references “26. World Health Organization. STEPS Manual, STEPS Instrument. Geneva: WHO; 2011, .” and “28. Kowal P, Chatterji S, Naidoo N, Biritwum R, Fan W, Lopez Ridaura R, et al. Data resource profile: the World Health Organization Study on global AGEing and adult health (SAGE). International journal of epidemiology. 2012;41(6):1639-49.” respectively. The WHO STEPS Manual does not provide the data set for the present study. Also the publication by Kowal P et al. seems to discuss WHO SAGE wave 0 and 1.
The authors can provide appropriate references for the stated methodology or outline them in detail in the current manuscript.

Thank you very much for these observations. This has been revised. The appropriate references for the study design have been added. In lines 87-92 (page 4), we have indicated that the data for this study are from the WHO Study on Global AGEing and Adult Health (SAGE) Wave 2 survey conducted in Ghana in 2015. The WHO SAGE aims to provide reliable evidence on the health and well-being of older persons aged over 50 years in low and middle-income countries [10]. The study design is provided elsewhere [11]. In brief, a stratified multistage cluster sampling method was used to collect data from a nationally representative sample of adults aged 50 years and older. Detailed descriptions of sampling methods and data collection procedures have been previously published [11-13].

2. The data availability statement also states that data is publicly available on the microdata repository of the WHO (<https://apps.who.int/healthinfo/systems/surveydata/index.php/catalog>). However, the data on this repository does not include the data used for the present study. If the data is available on specific request that should rather be made known by the authors.

Thank you very much for this observation. This has been revised. Data from SAGE Ghana Wave 2 was used for this study. The necessary permission was obtained from the World Health Organization to use the data. All files were obtained from the World Health Organization Study on global AGEing and adult health (WHO-SAGE). Details on data can be found at <http://www.who.int/healthinfo/sage/cohorts/en/>. The authors confirm that they had no special access privileges to the data. Interested researchers will have to submit a licensed data request to WHO. Upon approval, the researchers will be granted access to licensed data. Lines 375-380 (page 18)

3. Concerning the sample size and sampling:
I am curious to know what informed the authors’ decision to exclude 2,037 of the 3,575 original study sample because they had not used outpatient care in the 12 months preceding the survey; and why 12 months in particular? My thought is that, “not having used outpatient care” is a pattern of healthcare utilization, and this idea has completely been jettisoned. Frequency of hospitalization is independent of frequency of outpatient visit by their definition, so if persons with no outpatient visit in the preceding 12 months are excluded, essentially persons who may have been hospitalized in that period but had no outpatient visit are also excluded. If hospitalization is implied to be only after outpatient visit, then frequency of hospitalizations will be a subset of frequency of outpatient attendance. This seems to be a major flaw in sampling.

Thank you very much for this observation. This has been revised. In lines 93-103 (page 5), we have indicated that the original study sample comprised 3,575 older persons aged over 50 years. Participants were included in the current analysis if they had valid data on the key variables: chronic diseases such as angina pectoris, stroke, diabetes mellitus, hypertension, obesity, arthritis, asthma, chronic lung disease, depression, and cataracts and sociodemographic characteristics comprising sex, age, and employment. Participants (n=447) for which data on key variables were not captured or judged as invalid were excluded. Since the causes of missing information were not ascertained, we did not apply missing data techniques to avoid further uncertainty in the imputation models. Thus, the final analysis included 3,128 participants. Given the exclusion of 447 participants with missing data on the key study variables, the characteristics of the study participants with complete data were compared to those with incomplete data and no differences were found based on age, sex and employment status (See online Supplementary file 2).

4. On the measurement and definition of variables:
The frequency of hospitalization as defined is expected to have been reported as a continuous variable. However, in Table 2, it is categorized as “greater than or equal to 3” and by inference“1 to 2” outpatient visits. What informs this categorization at cut-off of 3? And the authors must at least state this categorization in the methodology. In the report of the results, line 186, it is interpreted as participants who visited outpatient clinic “more than three times” which is inaccurate. It should rather be stated as “three or more times”. The frequency of hospitalization as defined is expected to have been reported as a continuous variable. However, in Table 2, it is categorized as “Yes” and by inference “No”.
In the report of the results, line 186, it is interpreted as participants who were hospitalized at least once. This is not consistent with their methodology. The WHOQoL instrument gives results as a continuous variable(percentage between 0-100%). Can the authors explain why they categorized the results of QoL? …and in tertiles? Were the results normally distributed? Also why not as a binomial into poor and good quality of life because essentially the report lumped moderate and good quality of life as against poor quality of life in the results, lines 202-205 and discussion, lines 215-217. Can the result of QoL be reported or represented as odds of poor quality of life then.

Thank you very much for this important observations. This has been revised. In lines 112-121 (page 5) , we have indicated that the frequency of outpatient visits was measured as the number of times a participant had an outpatient visit in the preceding 12 months. Hospitalization was measured as any overnight stays in the hospital that lasted for at least one night in the past 12 months. An 8-item World Health Organization Quality of Life (WHOQOL) instrument was used to assess the QoL score [14]. The WHOQOL comprises two questions across each of the four main life domains: physical, psychological, social, and environmental [14]. Using a five-point Likert scale, ranging from very satisfied to very dissatisfied, the respondents rated their satisfaction with life domains such as health, ability to perform daily activities and meet basic needs, relationships, and environment. The composite score of QoL is the sum of the 8 items from the four domains expressed as a percentage.

5. History of medical conditions as well as screening questions for the chronic diseases were based on self-report which has been acknowledged as a major limitations of this study. Since hypertension was objectively screened for, the prevalence appears representative. Knowing that “hypertension and diabetes account for the highest burden of multimorbidity in Ghana (5) ” the prevalence of diabetes is expected to also be high, however, conditions that were not screened for like diabetes, stroke and cataract are likely to be under-reported.

Thank you very much pointing this out. Indeed one major strength of this study was the fact that screening for obesity, hypertension, angina pectoris, arthritis, asthma, chronic lung disease, and depression was based on objective measures comprising direct physical measurement of anthropometrics, BP, symptomatology algorithms and self-reports (lines 311-314 (page 15). However, under the limitation section (lines 318-320 ((page 15), we have indicated that the screening questions for diabetes, stroke and cataract were based on self-reported history of diagnosis. This may have resulted in the underestimation of the true prevalence of chronic diseases.

6. In the analysis:
The authors did not state the level of p-values at which analysed variables were considered statistically significant.
Thank you very much for this observation. The p-values at which analysed variables were considered statistically significant has been added (line 185, page 8).

Other minor issues:
7. The authors did not comply with using square brackets [] for citations. In Table 2, † did not appear in the legend.

Thank you very much for pointing out this. All the citations have been formatted and the legend for table 2 has been added

8. Under findings of latent class analysis:
What really is the meaning and import of “minimal cardiometabolic multimorbidity” in the present study? This should be addressed in the discussion as well. Were there any participants with no single morbidity or multimorbidity?

Thank you very much for this observation. This has been revised. Our findings show four distinct patterns of multimorbidity: relatively “healthy class” with minimal chronic diseases: abdominal obesity: cardiometabolic and arthritis class comprising participants with hypertension, diabetes, stroke, abdominal and general obesity, arthritis and cataract; and cardiopulmonary and depression class including participants with angina, chronic lung disease, asthma, and depression. (Lines 201-210 (page 9-10)).

9. In the discussion:
Line 235 to 236_ the study by Sum G et al.(22) as the authors cite, did not look at number of comorbidity only but also elucidated clusters of multimorbidities which (hypertension + arthritis) seemed to a predominant multimorbidity.

Thank you very much for this observation. This citation has been updated.

Line 239 to 241_ can the authors explain or give some examples of the several underlying mechanisms that could explain the associations in the present study?

Thank you very much for pointing out this. In lines 278-290 (page 14), we have indicated that the mechanisms that underlie the clustering of cardiopulmonary diseases and depression are not definitive. However, there is strong evidence linking inflammatory markers to both depression and cardiovascular diseases [7], but why these links exist remains unclear. Systematic reviews conducted by Mullerova et al. [8] and Prados-Torres et al. [9], identified inflammation, stress processes, hypoxia, and environmental risk factors such as air pollution and smoking as the leading risk factors for the clustering of cardiopulmonary diseases such as hypertension, angina, chronic lung disease and asthma [8, 9]. Similarly, our previous study on the patterns of cardiometabolic multimorbidity in sub-Saharan Africa identified the clustering of physical inactivity and obesity as one of the leading risk factors for cardiometabolic multimorbidity

10. In the references:
15. …Journal of Morbidity and Comorbidity not “Journal of Multimorbidity and Multimorbidity.”

Thank you very much for this observation. This citation has been updated.

**Reviewer 4**

Overall, this paper is well-written. This study identified three groups of patients with varying probabilities on nine diseases using a latent class analysis. The researchers also examined the associations between the class memberships and three outcome variables (outpatient visits, hospitalization, and QoL). The topic is important, and the results are interesting. However, there are some major comments or suggestions regarding the methods and results that could be considered to improve the quality of this paper.

Major:
1. Partial list of cardiometabolic diseases or conditions was included in this study. Other important diseases or conditions such as obesity, dyslipidemia, and chronic kidney disease were not included. If data on some or all of these diseases are available, it would be helpful to add them to the analysis. If not, it is suggested to discuss this as a limitation.

Thank you very much for this suggestion. We have revised the analysis and added general and abdominal obesity in the list of chronic diseases (figure 1 and 2). Under the limitation section (lines 329, page 17) we have indicated that the number of chronic diseases in the LCA was limited to those included in the SAGE survey in Ghana. This may have excluded other common chronic conditions among older persons, such as dementia, cancers chronic kidney disease, resulting in an underestimation of the multimorbidity burden. Future studies need to include more chronic diseases to increase the external validity

2. Because LCA is the main analysis in this study to identify latent classes of participants with multiple cardiometabolic diseases and non- cardiometabolic diseases, more details of results from the LCA models could be provided to help readers to understand the relationships between the sociodemographic and health characteristics and latent classes. For example, how were the nine disease variables coded and modeled in LCA? What is the distribution of the sociodemographic health characteristics of the study participants (as listed in Table 1) in the three latent classes?

Thank you very much for this observation. We have added a detailed description of the sociodemographic distribution of the multimorbidity classes identified in this study (Table 3, page 10). Further detail on the LCA and hierarchical cluster analysis has been provided in lines 147 to 172 (page 6 and 7).

3. What is the meaning of the beta coefficients from the negative binomial regression (Line 200-201)? What is the clinical significance of these results? This information is important for readers to interpret the findings.

Thank you very much for this observation. Further references on the interpretation of regression coefficients and odd rations have been added. In line 175, page 8, we have indicated that we used negative binomial regression to determine the association of multimorbidity combinations with outpatient visits. Negative binomial regression has inbuilt parameters that account for the overdispersion problem of modelling healthcare utilization frequency [15]. The association of multimorbidity combinations with hospitalization and QoL were assessed using multivariable logistic and linear regressions. The strength of association was interpreted using the adjusted odds ratios (aOR) and 95% confidence intervals (CI) from logistics regression and beta (β) coefficients from negative binomial and linear regressions [16, 17]. P values of <0.05 were considered statistically significant

4. How was QoL coded and analyzed in ordinal logistic regression? For example, was QoL coded as low = 0, moderate=1, and high=2 or vice versa in the ordinal logistic regression model? It is important to indicate this information in order to understand the direction and magnitude of the associations.

Thank you very much for this observation. This has been revised. In lines 112-121 (page 5) , we have indicated that the frequency of outpatient visits was measured as the number of times a participant had an outpatient visit in the preceding 12 months. Hospitalization was measured as any overnight stays in the hospital that lasted for at least one night in the past 12 months. An 8-item World Health Organization Quality of Life (WHOQOL) instrument was used to assess the QoL score [14]. The WHOQOL comprises two questions across each of the four main life domains: physical, psychological, social, and environmental [14]. Using a five-point Likert scale, ranging from very satisfied to very dissatisfied, the respondents rated their satisfaction with life domains such as health, ability to perform daily activities and meet basic needs, relationships, and environment. The composite score of QoL is the sum of the 8 items from the four domains expressed as a percentage.

Minor:
1. More details on the LCA modeling process could be added to the Method section (Line 134-142) to better assist readers in understanding how the latent classes were determined and how the participants were classified into each of the three latent classes. For example, the two sentences on Line 141-142 might be difficult to be understood by readers. What are the posterior probabilities? How are they related to the determination of class memberships?

Thank you very much for this observation. A reference on further details on the application of latent class analysis has been added [18]. In line 147-154, page 6-7, we have indicated that latent Class Analysis (LCA) was used to place participants in a number (K) of clinically meaningful classes of cardiometabolic multimorbidity. The number of multimorbidity classes was defined a priori using the adjusted Bayesian information criterion (BIC), a model selection method that balances fit with parsimony [18]. Five plausible LCA models were delineated, characterized by increasing numbers of chronic disease classes from one to five (Online supplementary file 2). The model with the lowest value of the BIC index was selected as the best-fitting model considering interpretability and clinical judgment [18, 19]. Posterior probabilities were used to determine the likelihood of class membership. Finally, the participants were grouped into the multimorbidity classes with the highest-class probability [20]. The multimorbidity classes are shown in Figure 1. In line 105-212, page 10, we have indicated that we compared LCA models with 1 to 5 classes (online supplementary file 2). The four-class model had the lowest BIC index and thus was selected as the best-fit model. Class one comprised relatively “healthy participants” with minimal chronic diseases (47.9%). Class two included participants with a high probability of abdominal obesity (40.7%). Class three comprised participants with high probabilities of hypertension, diabetes, stroke, abdominal and general obesity, arthritis and cataract (5.7%). Class four (cardiopulmonary diseases and depression) comprised participants with high probabilities of angina, chronic lung disease, asthma and depression (5.7%).

2. Reference # 26 indicates WHO STEP Manual 2011 (Line 380). However, data from the WHO Study on Global AGEing and Adult Health (SAGE) Wave 2 were used (Line 83).

Thank you very much for this observation. The reference has been revised and updated.

**References**

1.	Piette, J.D. and E.A. Kerr, *The impact of comorbid chronic conditions on diabetes care.* Diabetes care, 2006. **29**(3): p. 725-731.

2.	Kessler, R.C. and T.B. Üstün, *The world mental health (WMH) survey initiative version of the world health organization (WHO) composite international diagnostic interview (CIDI).* International journal of methods in psychiatric research, 2004. **13**(2): p. 93-121.

3.	Moussavi, S., et al., *Depression, chronic diseases, and decrements in health: results from the World Health Surveys.* The Lancet, 2007. **370**(9590): p. 851-858.

4.	Rose, G.A., *The diagnosis of ischaemic heart pain and intermittent claudication in field surveys.* Bulletin of the World Health Organization, 1962. **27**(6): p. 645.

5.	Organization, W.H. and I.S.o.H.W. Group, *2003 World Health Organization (WHO)/International Society of Hypertension (ISH) statement on management of hypertension.* Journal of hypertension, 2003. **21**(11): p. 1983-1992.

6.	Otieno, P., et al., *Multimorbidity of cardiometabolic diseases: A cross-sectional study of patterns, clusters and associated risk factors in sub-Saharan Africa.* BMJ open, 2023. **13**(2): p. e064275.

7.	Shao, M., et al., *Depression and cardiovascular disease: Shared molecular mechanisms and clinical implications.* Psychiatry research, 2020. **285**: p. 112802.

8.	Müllerova, H., et al., *Cardiovascular comorbidity in COPD: systematic literature review.* Chest, 2013. **144**(4): p. 1163-1178.

9.	Prados-Torres, A., et al., *Multimorbidity patterns: a systematic review.* Journal of clinical epidemiology, 2014. **67**(3): p. 254-266.

10.	He, W., M.N. Muenchrath, and P.R. Kowal, *Shades of gray: a cross-country study of health and well-being of the older populations in SAGE countries, 2007-2010*. 2012: US Department of Commerce, Economics and Statistics Administration, US ….

11.	Charlton, K., et al., *Leveraging ongoing research to evaluate the health impacts of South Africa's salt reduction strategy: a prospective nested cohort within the WHO-SAGE multicountry, longitudinal study.* BMJ open, 2016. **6**(11): p. e013316.

12.	Aheto, J.M.K., et al., *Prevalence, socio-demographic and environmental determinants of asthma in 4621 Ghanaian adults: Evidence from Wave 2 of the World Health Organization’s study on global AGEing and adult health.* Plos one, 2020. **15**(12): p. e0243642.

13.	Lartey, S.T., et al., *Evaluation of the association between health state utilities and obesity in sub-Saharan Africa: evidence from World Health organization study on global ageing and adult health wave 2.* Value in Health, 2019. **22**(9): p. 1042-1049.

14.	(WHO), W.H.O. *WHOQoL 8-items version*. [cited 2022 8/30]; Available from: <https://www.who.int/tools/whoqol>.

15.	Le, D.D., R.L. Gonzalez, and J.U. Matola, *Modeling count data for health care utilization: an empirical study of outpatient visits among Vietnamese older people.* BMC Medical Informatics and Decision Making, 2021. **21**(1): p. 1-14.

16.	Ali, P. and A. Younas, *Understanding and interpreting regression analysis.* Evidence-Based Nursing, 2021. **24**(4): p. 116-118.

17.	Peng, C.-Y.J. and T.-S.H. So, *Logistic regression analysis and reporting: A primer.* Understanding Statistics: Statistical Issues in Psychology, Education, and the Social Sciences, 2002. **1**(1): p. 31-70.

18.	Schwarz, G., *Estimating the dimension of a model.* The annals of statistics, 1978: p. 461-464.

19.	Akaike, H., *A new look at the statistical model identification.* IEEE transactions on automatic control, 1974. **19**(6): p. 716-723.

20.	Formann, A.K. and T. Kohlmann, *Latent class analysis in medical research.* Statistical methods in medical research, 1996. **5**(2): p. 179-211.
