# Supplementary material for: Cardiometabolic multimorbidity and associated patterns of healthcare utilization and quality of life: Results from the Study on Global AGEing and Adult Health (SAGE) Wave 2 in Ghana
Source: PLOS Glob Public Health. 2023 Aug 16;3(8):e0002215. doi: 10.1371/journal.pgph.0002215 (PMC10431646; doi:10.1371/journal.pgph.0002215)
Supplement: Attachment — Submitted filename: response-to-reviewers-comments.docx [file pgph.0002215.s004.docx]

**Journal Requirements:**

1.Please amend your detailed Financial Disclosure statement. This is published with the article. It must therefore be completed in full sentences and contain the exact wording you wish to be published. State what role the funders took in the study. If the funders had no role in your study, please state: “The funders had no role in study design, data collection and analysis, decision to publish, or preparation of the manuscript.”
b. If any authors received a salary from any of your funders, please state which authors and which funders. If you did not receive any funding for this study, please simply state: “The authors received no specific funding for this work.”

Thank you very much for this observation. This has been updated

**Additional Editor Comments:**

2. Lines 199-200: Was the higher prevalence of chronic lung diseases in women vs. men expected? What was this due to? Generally, in Africa, it’s higher in men because of their higher rates of smoking. I suggest that you rephrase this sentence and only report the conditions that are significantly different between the sexes.

Thank you very much for this observation. This has been revised. In lines 197-198, we have reported the conditions that are significantly different between the sexes.

3. Line 207: Please clarify what is meant by “minimal chronic diseases’ – is it no chronic diseases? Line 208: Does class two include abdominal obesity alone, with no other comorbidities?

Thank you very much for pointing this out. This has been revised. In lines 204-208, we have indicated that class one comprised relatively “healthy participants” with no multimorbidity. Class two included participants with a high probability of abdominal obesity. Class three comprised participants with high probabilities of hypertension, diabetes, stroke, abdominal and general obesity, arthritis and cataract. Class four (cardiopulmonary diseases and depression) comprised participants with high probabilities of angina, chronic lung disease, asthma and depression.

4. Please also clarify in table on Page 11. This should be Table 2 and not 3 – line 233. Please also correct table numbering on line 236 and 245.

Thank you very much for this observation. The table numbering has been updated

5. Table 1: I suggest presenting the column with the total (both sexes) data first followed by the male/female columns because the p-value relates to the latter columns. This will read better. P values in all tables should be to 3 decimal places, please.

Thank you very much for pointing out this. Table 1 has been revised. The demographic information for both sexes has been presented in the first column and the p-values adjusted to 3 decimal places

6. Line 298: Please add 'diseases' after 'cardiometabolic'. Line 322: Please add: ‘particularly for diabetes’ after ‘diseases'.

Thank you very much for these observations. This has been revised.

**Reviewers' comments:**

Reviewer #1: All comments have been addressed

Reviewer #3: All comments have been addressed

Reviewer #4: All comments have been addressed

Thank you very much reviewers 1, 3 and 4. Indeed, the findings of this study form the basis for, future research on the aetiology and pathogenesis of multimorbidity clusters, and improved policies to address healthcare access and quality of life for older persons living with cardiometabolic multimorbidity in sub-Saharan Africa.

7. Reviewer #4: Thank you for addressing the comments and suggestions raised on the previous version. The major revisions are appropriate and adequate. Adding the abdominal obesity variable to the LCA modeling is helpful. The results from the 4-class model are more meaningful than the previous results from the 3-class model. The results on the associations between latent class memberships and the healthcare utilization patterns and QoL have more clinical significance in this 4-class model compared to the previous 3-class model. The following are some additional comments and suggestions for you to consider. The results of the supplementary hierarchical cluster analysis seem to be unrelated to the objectives of the study and did not provide much support to the results of the LCA modeling either. They might cause unnecessary confusions to the readers. Suggest removing this method and the related results from the paper.

Thank you very much for this important observation. Although our goal was to define distinct clusters of multimorbidity using latent class analysis (LCA), we also investigated the extent to which these groupings remained stable following the latent class analysis. Thus we separately applied hierarchical agglomerative cluster analysis, a non–model-based cluster analysis. In general, our results were consistent with those obtained using LCA. The hierarchical clustering algorithms revealed distinct groupings of multimorbidity in the study sample. Based on the proximity coefficients, the first cluster comprised angina, chronic lung disease, asthma, and depression (cardiopulmonary and depression class). The second cluster comprised participants with hypertension, abdominal and general obesity, arthritis, cataract stroke and diabetes (cardiometabolic diseases, arthritis and cataract class). (Lines 202-208).

2. The labeling for latent class 3 and 4 seems not to reflect the highest probabilities of diseases within each latent class sufficiently. For example, in class 3, hypertension and abdominal obesity have the highest probabilities, followed by general obesity and arthritis. In class 4, asthma and chronic lung disease have the highest probabilities, followed by hypertension, abdominal obesity, and angina. As you show in Table 1, abdominal obesity has the highest prevalence, particularly in females. Hypertension has the second highest prevalence. Therefore, abdominal obesity and hypertension could be the two major diseases that could potentially be related to other comorbidities in this population. I would suggest using the following method to simplify the labeling of the four latent classes based on the major diseases with the highest probabilities within each latent class: class 1 - no comorbidity; class 2 - abdominal obesity; class 3 - cardiometabolic comorbidities; class 4 - cardiopulmonary comorbidities.

Thank you very much for these suggestions. We have adopted this suggestion and labelled the four latent classes; class 1 (relatively “healthy class” with no multimorbidity): class 2 (abdominal obesity): class 3 (cardiometabolic diseases and arthritis; and class 4 (cardiopulmonary diseases and depression) (Lines 202-208).
